# Integrating Proteomics into Personalized Medicine for Inflammatory Bowel Disease—Reality or Challenge?

**DOI:** 10.3390/ijms26114993

**Published:** 2025-05-22

**Authors:** Horia Minea, Ana-Maria Singeap, Manuela Minea, Simona Juncu, Stefan Andrei Chiriac, Catalin Victor Sfarti, Carol Stanciu, Anca Trifan

**Affiliations:** 1 Department of Gastroenterology, Grigore T. Popa University of Medicine and Pharmacy, 700115 Iasi, Romania; horia-octav.minea@umfiasi.ro (H.M.); ana.singeap@umfiasi.ro (A.-M.S.); andrei-chiriac@umfiasi.ro (S.A.C.); victor.sfarti@umfiasi.ro (C.V.S.); carol.stanciu@umfiasi.ro (C.S.); anca.trifan@umfiasi.ro (A.T.); 2 Institute of Gastroenterology and Hepatology, “St. Spiridon” University Hospital, 700111 Iasi, Romania; 3 Department of Microbiology, The National Institute of Public Health, 700464 Iasi, Romania; manuela.minea@insp.gov.ro

**Keywords:** inflammatory bowel diseases, proteomics, precision medicine, risk stratification

## Abstract

Inflammatory bowel diseases (IBD) represent chronic conditions with etiopathogenic mechanisms incompletely elucidated despite extensive research efforts. Therefore, it is essential for clinical monitoring of the implementation of personalized medicine, enabling risk stratification and the selection of therapies with the highest likelihood of a favorable response. Multi-omics approaches have emerged as an excellent opportunity for the prevention, clinical phenotype differentiation, and prediction of IBD development. Proteomics has gained significant enthusiasm in medical practice, primarily due to its focus on studying the composition and dynamic expression of various cellular and tissue structures. This approach provides critical insights into their impact on signaling pathways, post-translational modifications, and the development of sequence variations. Hence, it could provide the foundation for developing biomarkers with the potential to assess mucosal healing and predict prognostic variability among patients, facilitating the implementation of a personalized therapeutic approach. This review focuses on the recent research regarding the possibility of implementing proteomics technologies into clinical practice, given the challenges and limitations, and the advantages of increasing the quality of life in patients with IBD.

## 1. Introduction

Inflammatory bowel diseases (IBD) represent chronic conditions with a severe impact on patients’ quality of life and significant financial burdens on national healthcare systems, driven by a rising global trend in morbidity and mortality [1,2]. From 1990 to 2019, the number of individuals diagnosed with IBD increased significantly from 3.3 million to 4.9 million, while the number of associated deaths doubled, rising from 21.418 to 42.422 [3]. Until 2030, it is estimated that the number of cases of the disease in Europe and the United States will exceed seven million people [4].

Despite extensive research efforts, the etiopathogenic mechanisms associated with IBD remain incompletely elucidated. Studies have explored various contributing factors, including genetic predisposition, abnormal immune responses, environmental influences, and gut microbiome analysis, aiming to assess their impact on the development of interindividual variability [5].

One of the most important challenges in IBD management is the identification of biomarkers capable of accurately stratifying patients based on inflammation severity, disease progression, and the risk of relapse or complications. Hence, it is essential for clinical monitoring and the implementation of personalized medicine, enabling the selection of therapies with the highest likelihood of a favorable response [6,7].

Therefore, multi-omics approaches such as genomics, epigenomics, transcriptomics, proteomics, and metabolomics have emerged as an excellent opportunity for the prevention, clinical phenotype differentiation, and prediction of IBD. By simultaneously processing data on risk factors, exposure pathways, epithelial barrier function, gut microbiome variability, and cellular interactions mediating immune responses, multi-omics provides a multidimensional understanding of disease mechanisms [8,9,10].

In the past decade, the field of proteomics has advanced rapidly, enhancing the diversity of available information and leading to a more detailed understanding of the underlying pathogenic mechanisms [11,12,13]. Identifying patients as distinct individuals within a complex and diverse population is considered an essential approach to improving IBD management. Although IBD predominantly affects young adults, recently, the age of onset of the disease has been reported to be lower than 20 years for approximately 25% of patients. Therefore, these changes underscore the necessity for enhanced research focused on establishing new biomarkers. Moreover, they would allow for early detection and aid in selecting suitable treatment options [14].

The discovery and characterization of possible molecular targets involved in the pathogenesis of the disease have improved the performance of investigations and allowed precise identification of subtypes associated with susceptible individuals or those presenting a subclinical, potentially reversible form [15,16]. Another explored priority represents the non-invasive follow-up of endoscopic or histological activity to anticipate adverse events associated with certain medications and the necessity of treatment adjustment [17,18].

Based on the integration of data obtained with these technologies, reference maps have been designed to present an increased level of precision and cellular or molecular interactions at the level of different normal or inflamed tissues. These findings could serve as specific signatures for unraveling the complexity of these diseases and defining new therapeutic targets [19]. A significant contribution to understanding the various pathological pathways in the initiation and progression of IBD was made by genomic and transcriptomic studies [20]. However, it has been noticed that genetic alterations do not always translate into clinical phenotypes. Instead, proteomic research could serve as the link between the genome, transcriptome, and phenotypic representation of these diseases [21].

Proteins represent essential functional units of the human organism, playing a significant role in most intracellular physiological processes and intercellular interactions. Proteomics has gained significant enthusiasm in medical practice, primarily due to its focus on studying the composition and dynamic expression of various cellular and tissue structures. This approach provides critical insights into their impact on signaling pathways, post-translational modifications, and the development of sequence variations [22].

Numerous studies have been widely explored to investigate differences in clinical behavior, and the analysis of proteomic profiles has advanced significantly in recent years. Critical insights into the molecular mechanisms underlying IBD pathogenesis have provided the foundation for developing biomarkers with the potential to assess mucosal healing and predict prognostic variability among patients, facilitating the implementation of a personalized therapeutic approach [23].

The main advantage of these technologies focuses on their ability to provide detailed molecular insights into the regulatory mechanisms involved in human physiological and pathological processes [24]. However, detecting these compounds remains a significant challenge due to the diversity of their physicochemical properties and the narrow concentration ranges in biological samples. An increased number of methods of detection are available, each one presenting advantages and challenges regarding applicability, sensitivity, and reproducibility [25].

Among the high-resolution techniques used to discover new targets, two-dimensional polyacrylamide gel electrophoresis (2-DE) stands out for its ability to separate and quantify proteins on the same gel according to isoelectric points and molecular weights, with the potential to additionally perform a complex analysis of isoform structures and post-translational changes [26]. Even though it is valuable for answering various clinical questions, its implementation in practice is limited because the accuracy and precision represent a challenge for ensuring the reproducibility of results [27]. In contrast, two-dimensional difference gel electrophoresis (2D-DIGE) has a much better yield and provides reliable results that are associated with specific signals obtained by fluorescently labeling proteins [28]. While 2DE and 2D-DIGE allow for the separation of large amounts of proteins, mass spectrometry (MS) has become a frequently used tool for comparing their relative expression levels in different samples. The main benefit of the accelerated development of quantitative proteomic techniques based on MS is the stimulation of the discovery of new biomarkers or therapeutic targets [27,29].

The use of stable isotopes for in vivo labeling of amino acids (SILAC) has been shown to considerably reduce the variability of the results. However, it is only applicable in studies using cell cultures or model organisms because it requires the cultivation of samples in special media for the incorporation of stable isotopes during growth. Another disadvantage of SILAC refers to the possibility of comparing only 2–3 samples [30].

The first approach implemented for in vitro chemical labeling is associated with the application of labels containing thiol-reactive groups for the incorporation of stable isotopes (ICAT). After digestion of the labeled proteins, only the cysteine-containing peptides are enriched by affinity chromatography and subsequently quantified by MS. However, the use of ICAT in medical practice is limited because the proteome coverage is reduced, being oriented towards the exclusive analysis of cysteine-containing proteins [27].

Capitalizing on the advances in MS, various technologies based on in vitro isobaric labeling with Tags for Relative and Absolute Quantitation (iTRAQ) or with Tandem Mass Tags (TMT) with stable isotopes have been developed. Due to the improvement in the data analysis algorithm and the optimization of the workflows for the simultaneous combination and quantification of peptides from multiple samples, they have become extremely popular, being associated with an increase in throughput and a reduction in experimental variability. Despite the flexibility and the ability to multiplex up to 16 samples, the attractiveness of using these techniques has been undermined by variations in ion intensity and spectral quality that could affect the accuracy of quantification, especially of low-abundance peptides or complex samples [31,32].

In label-free methods, peptides are quantified based on spectral intensity and counting (LQR) or based on chromatographic peak integration (LC) that identifies peptides by MS. Unlike stable isotope techniques, label-free approaches have much lower precision and reproducibility because unsystematic variations influence MS results [29]. However, there are several advantages. First, there is no limit to the number of samples that could be compared in an experiment. Second, it offers the possibility of dynamic quantification of protein expression [33].

Of the advanced methods, Sequential Window Acquisition of All Theoretical Mass Spectra–Mass Spectrometry (SWATH-MS) and Matrix-Assisted Laser Desorption/Ionization Time-of-Flight Mass Spectrometry (MALDI-TOF-MS) offer extensive and reproducible proteomic coverage, enabling precise monitoring of molecular variations in tissues and biological fluids [27,34]. Table 1 presents the characteristics of the most important techniques used in the detection process of proteomics.

**Figure 1:**
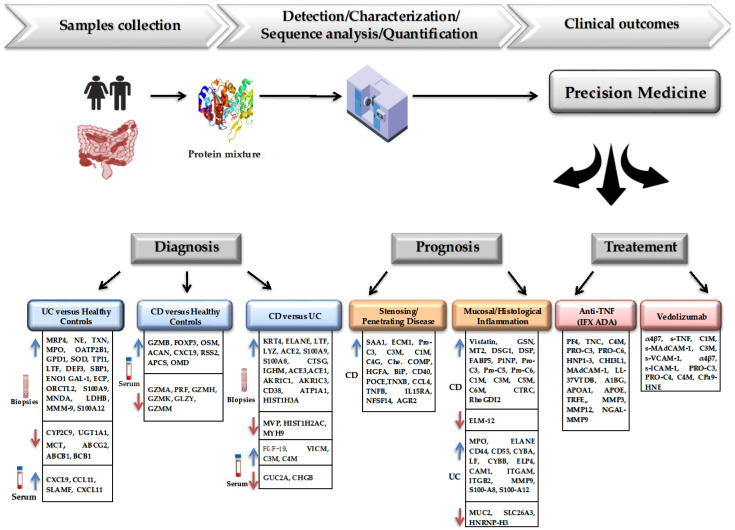
Potential applications of proteomics for implementing precision medicine in IBD. The first section focuses on the biomarkers involved in the differential diagnosis, including proteins capable of distinguishing IBD patients from controls, as well as CD from UC. The middle section presents the prognostic stratification, identifying protein signatures that could predict complications such as stenosis, penetrating behavior, and biomarkers that could correlate with the mucosal and histologic activity of the disease. The last compartment highlights the predictors of treatment response, where distinct proteomic patterns are associated with favorable or non-favorable outcomes following biological therapy.

Using these methods, valuable insights have been obtained, providing a detailed perspective—from the involvement of genetic and non-genetic risk factors to the detection of molecular alterations associated with various pathological conditions [35]. Therefore, in this review, we highlighted the advancements made in recent years in the field of diagnostic, prognostic, and response biomarkers, emphasizing the limitations, challenges, and opportunities associated with the application of proteomic technologies. These approaches aim to enhance the understanding of the molecular mechanisms underlying IBD pathogenesis and enable risk-based patient stratification, ultimately improving clinical and therapeutic management (Figure 1).

## 2. The Role of Proteomics in Diagnosis and Susceptibility to IBD

The dynamic distribution of protein macromolecules ensures the maintenance of internal homeostasis. Hence, analyzing subcellular organization is crucial for bridging the gap between the genetic profile and clinical phenotypes [36]. Investigating protein interactions and post-translational expression levels represents a complex approach with an increased potential for understanding the pathogenic mechanisms of the diseases by highlighting the connection between genomic or transcriptomic alterations and the expression of the clinical phenotype [21,37].

Alterations in certain peptide sequences could influence various cellular transport pathways, leading to dysfunctions associated with the onset of these diseases. In ulcerative colitis (UC), cytoskeletal rearrangement occurs, resulting in the release of elevated levels of enzymes such as glycerol-3-phosphate dehydrogenase, tricarboxylic acid cycle enzymes, oxidative phosphorylation enzymes, and carbonyl reductase, which are involved in sustained intestinal inflammation [38].

Advancements in the development of proteomic techniques provide multiple opportunities for identifying molecular signatures associated with different disease progression states in IBD [39].

Unlike traditional immunoassay techniques, which focus on detecting a single biomarker, the identification of a broad proteomic spectrum provides a more accurate representation of physiological and clinical variations. This approach enhances disease activity monitoring, relapse prediction, and treatment response assessment [40,41,42].

The application of new examination methods has enabled the identification of molecular alterations in junction proteins and the extracellular matrix within inflamed tissue, providing insights that have enhanced the understanding of IBD pathogenic mechanisms [39].

The benefit of using serum proteomes to define clinical behavior was evaluated by Kalla et al. in a multicenter study involving a cohort of 552 IBD patients. Out of 313 protein markers analyzed, 66 were confirmed for their ability to differentiate IBD patients from the control group. Additionally, they identified 55 differentially expressed protein markers in CD, with the most significant being chemokine ligand 9 (CXCL9) and Oncostatin M (OSM). In contrast, for UC, they reported significant differences compared to controls in only 46 proteomes, primarily matrix metalloproteinase-12 (MMP-12) and Granzyme B (GZMB) [43].

A major objective in IBD management is the early identification of individuals with increased susceptibility, allowing for the implementation of effective preventive strategies and symptom mitigation. The concept of a preclinical phase in these diseases was suggested by findings from the PREDICTS study. Using a signature of 51 serum proteins, the imminent development of CD within the next five years was associated with alterations in the complement activation cascade, lysosomal regulation, innate immune response, and glycosaminoglycan metabolism. These findings confirm that various biological processes are activated long before the clinical diagnosis is established [44].

On the other hand, Bergemalm et al. demonstrated the involvement of six inflammatory serum proteins, matrix metalloproteinase-10 (MMP10), chemokine ligand 9 (CXCL9), CC motif chemokine ligand 1 (CCL11), SLAM family member 1 (SLAMF1), C-X-C motif chemokine ligand 11 (CXCL11) and monocyte chemoattractant protein-1 (MCP-1) that are upregulated before the onset of UC compared to healthy controls. Among these, MMP10, CXCL9, CXCL11, and MCP-1 stood out as they remained consistently elevated even among healthy twin siblings [45]. Table 2 provides a summary of proteins and pathways identified through the proteomic approach as being involved in the pathogenesis of IBD that could serve as diagnostic predictors.

## 3. The Role of Proteomics as a Prognostic Factor in the Evolution of IBD Patients

Using a panel of 13 serum proteins, the Endoscopic Healing Index (EHI) was defined. This index validated the prediction of remission in CD patients, demonstrating accuracy comparable to fecal calprotectin (FC) and significantly superior to C-reactive protein (CRP) [58].

A recent study highlighted the involvement of a wide range of differentially expressed proteins in the feces of IBD patients compared to controls, with a predominant upregulation of immunoglobulins and neutrophil-associated proteins. Additionally, the underexpression of Olfactomedin-4 (OLFM4), Ectonucleotide Pyrophosphatase/Phosphodiesterase (ENPP), or nucleic acid-associated proteins was observed to increase the cancer risk in these patients [59]. Lucaciu et al. proved that 24 proteins associated with the acute inflammatory response exhibit distinct signals when comparing CD to controls. Among these, WD repeat-containing protein 31 (WDR31), alpha-2-glycoprotein rich in leucine (LRG1), and serum amyloid A1 (SAA1) were notably abundant in patients with a stricturing or penetrating phenotype [37].

Regarding the relapse of the disease, in a study monitoring CD patients in clinical remission, an increased risk of a flare in the short term (<6 months) was observed in relation to the presence of a specific combination of 15 proteins. This panel includes acute-phase reactants, complement components (C3, C4B, C5, CFH—Complement Factor H, CFHR2—Complement Factor H-Related Protein 2), coagulation-related proteins (F9—Coagulation Factor IX, SERPIND1—Heparin cofactor II), metal-binding or transport proteins (CP—Ceruloplasmin, HPR—Haptoglobin-related protein), lipid metabolism and apolipoproteins (APOC4—Apolipoprotein C-IV), mannose-binding lectin pathway (MBL2), extracellular matrix or inter-alpha-trypsin inhibitors (ITIH2, ITIH3), and inflammation-associated proteins (LRG1–Leucine-rich alpha–2- glycoprotein 1, APCS—Serum amyloid P-component) [60].

Bennike et al. identified a specific signature in the biopsies of **UC** patients, corresponding to a significant increase in 46 proteins with various biological and molecular functions. Additionally, it was noticed that 11 of these proteins Lactotransferrin (LTF); Matrix Metalloproteinase-9 (MMP-9); Myeloperoxidase (MPO); Neutrophil Elastase (NE); Protein S100-A9 (S100A9); Protein S100-A12 (S100A12); Neutrophil Defensin 3 (DEFA3); Galectin-10 (Gal-10); Eosinophil Cationic Protein (ECP); Myeloid Cell Nuclear Differentiation Antigen (MNDA); Cathepsin G (CTSG) are frequently detected in polymorphonuclear neutrophils (PMN). Moreover, they play a crucial role in forming extracellular traps, from which they are released in response to immune stimuli. Even in the absence of visible changes in the colonic mucosa, the authors suggested that the abundance of these proteins could be considered a pathognomonic marker of chronic inflammation, a finding confirmed by confocal laser endomicroscopy (CLE) [41].

Using high-resolution mass spectrometry, differentially expressed proteins were quantified in Th1 and Th1/Th17 clones isolated from the intestinal mucosa of **CD** patients. The primary difference observed between these two phenotypes was related to the expression of specific cytotoxic proteins—Granzyme B (GZMB), Granzyme K (GZMK), Granulysin (GNLY), and Perforin (PRF1)—which were detected in high amounts in Th1 cells. In contrast, transcription factors of CD4+ cells involved in inflammatory or immunosuppressive responses were predominant in Th1/Th17 clones [61].

It is widely recognized that IBD exhibits significant phenotypic variability related to disease localization and clinical prognosis, which has a major impact on selecting an appropriate treatment. One example is the abundance of a panel consisting of five serum glycoproteins—Cartilage Oligomeric Matrix Protein (COMP), Hepatocyte Growth Factor Activator (HGFA), Procollagen C-Endopeptidase Enhancer (POCE), Cholinesterase (Che), and Tenascin-X (TNXB)—which states a significant role in predicting the development of stricturing complications in CD patients compared to the inflammatory phenotype [62].

Clinical, endoscopic, and histologic monitoring of inflammatory intestinal lesions represents essential factors in the management of IBD, having a crucial role in selecting a treatment that promotes deep remission and prevents the development of severe complications [63].

In recent years, histological activity assessment has been considered the most reliable method for accurately stratifying patients based on the likelihood of relapses or severe complications, which leads to increased hospitalization and surgical intervention rates. However, numerous perspectives have emerged that support the potential of modern proteomic technologies as an additional tool that could be implemented to identify these risks [64]. Hence, Gruver et al. identified a strong correlation between the severe prognosis of **UC** patients, assessed using the Geboes Score or the Robarts Histopathology Index (RHI), and neutrophil-associated proteins. Additionally, the study reported an inverse relationship between these scores and cell junction proteins as well as β-catenin, most likely resulting from the disruption of intestinal crypt architecture [65].

The advancements in proteomic technologies in recent years, driven by the incorporation of stable isotopes, have enabled the quantitative profiling of various biological structures. As a result, numerous molecular alterations have been identified in the extracellular matrix, metabolic reprogramming, and autophagy within intestinal epithelial cells, contributing to a deeper understanding of the complex mechanisms underlying IBD. Compared to healthy controls, **UC** patients exhibit a decrease in fatty acid synthase and increased levels of heavy-chain **p62 protein**, both of which are involved in autophagy [53].

In parallel, a study investigating colonic biopsies from children with IBD highlighted the role of a group of five proteins—Fatty acid-binding protein, epidermal (FABP5), Nicotinamide phosphoribosyltransferase (NAMPT/Visfatin), UDP-glucose 6-dehydrogenase (UGDH), Leucine-rich pentatricopeptide repeat motif-containing protein, mitochondrial (LRPPRC), and Inorganic pyrophosphatase (PPA1)—in distinguishing affected individuals from healthy controls. Additionally, the authors proposed a panel of 12 proteins as a candidate biomarker for differentiating CD from UC, including Hydroxyacyl-CoA dehydrogenase trifunctional multienzyme complex subunit beta (HADHB), Protein transport protein Sec61 subunit alpha isoform 1 (SEC61A1), Staphylococcal nuclease domain-containing protein 1 (SND1), Leucine aminopeptidase 3 (LAP3), Leukotriene A-4 hydrolase (LTA4H), Metallothionein-2 (MT2), Solute carrier family 25 member 1 (SLC25A1), Heterogeneous nuclear ribonucleoprotein H3 (HNRNPH3), Serotransferrin (TF), Delta(3,5)-Delta(2,4)-dienoyl-CoA isomerase (ECH1), Transferrin receptor protein 1 (TFRC), and Beta-2-microglobulin (B2M). In the same context, it was noticed that IBD patients had lower levels of fatty acid-binding protein 5 (FABP5), directly associated with alterations in compounds involved in energy metabolism, including pyrophosphatase, visfatin, and UDP-glucose 6-dehydrogenase, as well as disease severity. Furthermore, the results stated that the upregulation of proteins involved in fatty acid metabolism—hydrolase, tricarboxylate transport protein, trifunctional enzyme, and delta(3,5)-delta(2,4)-dienoyl-CoA isomerase—was specific to CD patients [51].

Another perspective on discovering the molecular mechanisms involved in IBD pathogenesis is related to the detection of an abundance of cell adhesion proteins, such as CD38, along with increased levels of angiotensin-converting enzymes 1 and 2, which have a key role in blood pressure regulation in CD patients [50]. On the other hand, it has been discovered that a significant factor in the progression of UC is the alteration in mucus composition, which enhances intestinal barrier permeability. This is due to reduced levels of the anion exchanger solute carrier family 26 member 3 (SLC26A3), which supplies bicarbonate to the apical membrane, as well as a decreased number of sentinel goblet cells [66]. There is evidence suggesting that various proteomic signatures detected in colonic biopsies could be recommended not only for differentiating CD from UC, but with greater accuracy compared to circulating cytokines proposed for stratifying IBD patients [53,67]. Table 3 provides a summary of proteins and pathways identified through the proteomic approach that could be monitored in order to anticipate the evolution of the disease in IBD patients.

## 4. Application of Proteomics in Optimizing Treatment Approaches for IBD

The selection of an effective therapeutic strategy is influenced by the intensity and extent of intestinal inflammatory lesions, while the maintenance or escalation phase primarily depends on the patient’s response. However, it is stated that only one-third of **IBD** patients achieve remission. Hence, there are insufficient data regarding the stratification of patients at high risk of relapses or those who exhibit a primary non-response to treatment [76,77]. Due to the increased number of IBD patients who develop intolerance or experience a loss of response over time, predicting treatment outcomes represents an essential factor in order to implement a personalized management approach that prevents progression to severe complications [78].

Therefore, an innovative field of study represents the discovery of the significant potential of proteomes as biomarkers for predicting clinical response to biological drugs. This could allow the implementation of precision medicine, in which each patient receives a therapy adapted to their individual profile. Hence, the rational use of resources is ensured, and excessive administration of medications to which patients have not responded favorably could be avoided. However, only a reduced number of studies have evaluated in recent years the potential of applying proteomics for predicting the response to different treatments administered in IBD patients [18].

Most proteomic studies have evaluated the patients treated with infliximab (IFX) [79,80]. Recently, the study conducted by Winter et al. addressed the complex pharmacokinetic behavior of the exposure–response relationship for anti-TNF, marked by the intervention of proteolytic factors that influence clearance and degradation. The authors identified nine proinflammatory proteins involved in increased intestinal permeability and worsening clinical course, which had significantly increased concentrations in non-responding IBD patients [81].

In the same context, there is evidence confirming the involvement of increased concentrations of matrix metalloproteinases (MMP3 and MMP12) in the inflamed intestinal mucosa being involved in the decrease in the integrity and function of infliximab (IFX) and adalimumab (ADA), which contributes to the establishment of a negative therapeutic response [82].

Gazouli et al. examined the serum of IBD patients before and after the introduction of IFX treatment. They were able to identify 15 differentially expressed proteins, the majority involved in immune reactions, such as apolipoprotein AI (APOA1), apolipoprotein E (APOE), complement C4-B (CO4B), plasminogen (PLMN), serotransferrin (TRFE), β-2-glycoprotein 1 (APOH), and clusterin (CLUS) that intervene in monocyte/macrophage activation or CD4+ T lymphocyte regulation and differentiation. These proteins were upregulated in the serum of patients who achieved clinical remission, with no differences in non-responders. Instead, a particularity reported for responders is related to increased levels of alpha-2-leucine-rich glycoprotein (A2GL), vitamin D binding protein (VTDB), alpha-1B-glycoprotein (A1BG), and C1r, an essential component of the complement (C1R) [83]. Understanding the pathogenic mechanisms involved in the primary lack of response stands as the main objective addressed in the first pilot study that analyzed the proteomic profile of CD patients before the initiation and after the completion of the induction period with IFX. The authors defined a model that included four serum biomarkers of platelet metabolism, the most important being platelet aggregation factor four (PF4), with much higher concentrations detected in those who did not have a favorable response compared to those in whom IFX administration induced remission [84]. In UC patients, the lack of a favorable response after two weeks of IFX therapy initiation is influenced by the increased reactivity of monocytes to inflammatory stimuli upon reaching the intestinal mucosa. In contrast, responders exhibit a significant reduction in tissue concentrations of Tenascin C (TNC) compared to baseline levels, which inhibits the release of C-C motif chemokine ligand 2 (CCL2) from inflammatory and stromal cells [85].

Although IFX represents one of the most widely used medications in the field of IBD, the factors predicting the response and the molecular mechanisms associated with loss of efficacy over time or primary non-responsiveness are not completely understood. As an example, TNC is an extracellular matrix glycoprotein synthesized in response to inflammation and tissue injury. In patients with a lack of response to infliximab, pre-treatment analysis revealed TNC overexpression in the lamina propria of the intestinal mucosa, which promotes the synthesis of a proinflammatory cytokine, interleukin-6 (IL-6), in human monocytes [50].

The intestinal extracellular matrix (ECM) represents a dynamic and multifunctional structure composed of a complex network of proteins that has an essential role in maintaining epithelial integrity and modulating the expression of clinical phenotypes [86]. It has been observed that the alteration in the remodeling balance of this structure due to the intensification of protein catabolism causes the extension of tissue lesions in CD. This favors the accumulation of inflammatory cells that initiate excessive ECM repair and remodeling processes that lead to the formation of fibrostenotic strictures. Therefore, increased amounts of degradation proteins and formation fragments of the ECM are released and could be considered serum biomarkers that reflect the evolution of the pathological process of the disease [87].

Moreover, the accumulation of type IV collagen (C4M) in the intestinal tissue is noted, which maintains chronic inflammation and increases the permeability of the intestinal mucosa in CD patients [88].

Furthermore, Alexdottir et al. concluded that serum C4M turnover could accurately stratify CD patients with a history of surgery into responders versus non-responders before initiating IFX administration, while biomarkers of collagen type III (PRO-C3) and VI (PRO-C6) formation might be used to monitor therapeutic efficacy [89]. There is evidence supporting the efficacy of IFX or ADA treatment for inducing and maintaining clinical and endoscopic remission in CD [90]. However, it has been reported that approximately 30% of these patients did not respond or had insufficient improvement after 14 weeks of anti-TNF-α induction. In this context, as further medications become available, the necessity to identify biomarkers that predict response for changing therapy or recommending surgery has increased [91].

Recently, the examination of a cohort of patients with UC revealed 257 proteins with differential expression in responders and non-responders to IFX. After excluding those caused by the severity of inflammation, only ß-actin-like protein 2 (ACTBL2), mannose-binding protein C (MBL2), bactericidal permeability-increasing protein (BPI), and eukaryotic translation initiation factor-3 (EIF3D) were validated as potential biomarkers of non-responsiveness to IFX therapy [92].

Among the 66 serum proteins that allowed the differentiation of IBD patients from non-IBD controls in the multicenter study conducted by Kalla et al., a panel was noted that included integrin alpha-V (ITGAV), epithelial cell adhesion molecule (EpCAM), IL-18, IL-8, and SLAM family member 7 (SLAMF7), with which a risk stratification was performed to select individuals requiring escalation of biological therapy or surgical intervention [43]. Table 4 presents the most studied predictive biomarkers in IBD therapy identified through proteomic research.

## 5. Limitations of Proteomics Study in IBD

Although these technologies of protein detection are extremely complex, they are still not capable of fully quantifying the protein structures present in a sample due to the diversity of chemical properties (polarity, charge, molecular weight, and stability [101]. Another aspect that decreases the prospects for implementing proteomics in medical practice refers to the technical challenge and increased costs that require a reduction in the size of the batches used to validate the accuracy of these biomarkers [20,102].

In addition to the technical barriers mentioned above, ensuring the reproducibility of results depends largely on the selection of representative samples that reflect the real dynamics of structural composition changes and, especially, on the use of methods with increased performance of detection that could eliminate the risk of masking proteins with a reduced level by high-abundance ones [29,103].

However, a persistent obstacle refers to insufficient multimodal integration of data due to heterogeneity and, above all, the lack of standardization criteria for achieving an objective interpretation that restricts the possibility of clinical translation of the various protein sequences used for evaluations at the level of molecular structures [7,104].

A crucial role in harnessing the potential of the proteomic approach is related to the necessity to use informatics tools capable of achieving spatial integration of datasets to assess the significance of the results, depending on the biological variability recorded between patient samples and the impact of environmental factors [11].

Although the potential of proteomics for evaluating treatment outcomes in IBD is recognized, there are opinions that argue that individual efficacy is influenced by the complex connection between the genome, microbiome, lifestyle, and environmental factors [105]. In this context, the integration of proteomic sequences with other omics data and clinical information is required to validate biomarkers with superior performance and predict therapeutic responses in these patients [106].

## 6. Conclusions and Future Directions

Since patients with IBD have come to be frequently diagnosed at young ages, and the clinical evolution is not limited to just a few years, it is necessary to intensify research to define biomarkers that would allow for detection in early stages and the selection of targeted therapy, considering the effectiveness of the response or the occurrence of potential adverse effects.

Approaching the patient as an individual entity in a complex and heterogeneous population is considered the optimal approach for achieving success in the management of these diseases. The accelerated development of proteomic techniques has improved the quality of information, achieving a detailed characterization of the human body at the molecular and cellular level that has made an important contribution to deciphering pathogenic mechanisms.

Compared to genomics and transcriptomics, this field explores post-translational modifications of proteins, providing a detailed picture of inflammatory processes and the intervention of the immune response in IBD. The advantage of applying these technologies refers to the possibility of integrating an increased number of variables to form a spatial reference map of complex interactions at the level of different normal or inflamed tissues, which facilitates a much more precise intervention compared to investigating the disruption of a single element.

In order to implement precision medicine in IBD, further research is needed to validate multiple proteomic panels as substitutes for simple biomarkers, which are capable of providing support both for improving the ability to understand the complexity of inflammatory disorders and for identifying risk stratification strategies that allow for optimizing the selection of therapeutic regimens.

## Figures and Tables

**Table 1:** Main Characteristics and Limitations of Proteomic Technologies Applied in IBD.

Type of Technology	Principle	Advantages	Disadvantages	References
2DE	Proteins are fractionated and separated based on their isoelectric point and molecular weight on polyacrylamide gels.	Low cost. High resolution. Able to analyze complex structures, including protein isoforms and post-translational modifications.	Biological variation—low reproducibility. Laborious. Protein identification requires additional MS.	[26,28]
2D-DIGE	Uses spectrally distinct fluorescent dyes for sample labeling, allowing comparative analysis of up to three proteins on a single gel.	Higher sensitivity, accuracy, and reproducibility. Increased detection rate compared to 2DE.	Laborious. Protein identification requires additional MS.	[26]
SILAC	Normal and heavy stable isotope labeling by amino acids in cell culture enables precise quantification of protein abundance.	Simple. Accurate and reproducible quantification. Suitable for dynamic studies of protein turnover.	Limited to cell culture systems or model organisms. Not applicable to complex biological samples. Samples must be grown in custom media to incorporate stable isotopes during growth.	[30]
SILAMi	Isotopically labeled microorganisms are used to trace protein synthesis in microbial communities.	Allows study of microbial dynamics and interactions. Suitable for intestinal microbiome-related proteomics.	Requires specific growth conditions for labeled microorganisms. Limited application for clinical investigations.	[29]
ICAT	Using a reagent that contains a reactive group towards thiol groups, a linker to incorporate stable isotopes (2H/1H), and an affinity tag to isolate isotope-labeled proteins/peptides (chemical labeling in vitro).	High accuracy for quantitative proteomic analysis of cells and tissues.	Requires chromatographic separation techniques.	[28]
iTRAQ	Proteins are digested into peptides, which are labeled with isobaric tags. Quantification is achieved by measuring reporter ion intensities during mass spectrometry analysis (chemical labeling in vitro).	Increased multiplexing capability. Enables relative and absolute quantification of proteins in multiple samples.	Complex sample preparation. Requires advanced MS expertise.	[31,32]
TMT	Proteins are divided into peptides and labeled with TMT reagents that release reporter ions during MS2 for quantification (chemical labeling in vitro).	Enables multiplexing of up to 16 samples. Suitable for comparative proteomics.	Expensive reagents. Potential interference between reporter ions at high multiplexing levels. Quantitative precision is dependent on the reproducibility of sample preparation.	[32]
LFQ	Peptides are quantified based on MS signal intensity or spectral counting without additional labeling.	Simple sample preparation. Supports high-throughput proteomic analysis. Dynamically detects differential protein expression.	Less accurate than the labeling methods (TMT). Reduced repeatability. The stability of experimental operation is demanding. Low reproducibility. Additional time needed for MS analysis.	[33]
LC-MS/MS	Chemical compounds are separated by liquid chromatography and analyzed by mass spectrometry to identify and quantify proteins.	High sensitivity and versatility. Capable of analyzing a wide range of biomolecules with high resolution.	Incomplete protein digestion. Difficulties in chromatographic separation of peptides. Requires advanced instrumentation and expertise. High operational costs.	[25]
MALDI-TOF-MS	Proteins or peptides are ionized using a laser, and their mass-to-charge ratios are analyzed in a time-of-flight mass spectrometer.	Rapid analysis of biomolecules. Minimal sample preparation. Suitable for high-throughput proteomic studies.	Limited sensitivity for low-abundance proteins. Lower resolution compared to other MS techniques.	[27]
SWATH-MS	A data-independent acquisition method where all precursor ions within a defined mass range are fragmented systematically and analyzed simultaneously.	Low-cost. High sensitivity and comprehensive coverage. Allows quantification of thousands of complex proteomes in a single run.	Requires advanced instrumentation and data analysis software. Computationally intensive and time-consuming for processing large datasets.	[34]

2DE: two-dimensional gel electrophoresis; 2D-DIGE: two-dimensional difference gel electrophoresis; SILAC: Stable Isotope Labeling with Amino Acids in Cell Culture; SILAMi: Stable Isotope Labeling of Microorganisms; ICAT: isotope-coded affinity tags; iTRAQ: Isobaric Tags for Relative and Absolute Quantification; TMT: tandem mass tag; MS: Mass Spectrometry; LFQ: Label-free quantification; LC-MS/MS: Liquid chromatography-mass spectrometry; MALDI-TOF-MS: Matrix-Assisted Laser Desorption/Ionization Time-of-Flight Mass Spectrometry; SWATH-MS: Sequential windowed acquisition of all theoretical fragment ions-mass spectrometry.

**Table 2:** Proteomics as diagnostic biomarkers in IBD.

Disease	Number of Patients	Sample	Proteomic Model		Main Findings	References
UC	72 patients versus 140 controls	Plasma	MMP10, MCP-1, CXCL9, CCL11, SLAMF1, CXCL11	Increased	The model was upregulated before the onset of UC compared to healthy controls (AUC = 0.92, *p* < 0.05).	[45]
UC	10 patients versus 10 controls	Colonic tissue	LTF, NE, ECP, MMP-9, MPO, MNDA, CatG, S100-A9, Gal-10, S100-A12, DEF3	Increased	High abundance in neutrophils (on average 42.2 times, *p* < 0.005) is associated with NETs formation in UC compared to controls. The severity of histological inflammation correlates with LTF (*r* = 0.91) and S100-A9 (*r* = 0.82, *p* < 0.001).	[46]
UC	10 patients versus 10 controls	Colonic tissue	MRP4, ORCTL2, OATP2B1	Increased	Significant modification of the expression profile of metabolizing enzymes and protein transporters in inflamed tissue in UC patients compared to controls.	[47]
			ABCB1, MCT1, ABCG2,	Decreased		
UC	55 patients versus 7 controls	Colonic tissue	CD47, NDUFAF4, AGPAT1, LSM-7, TMEM192	Increased	Five proteins are differentially expressed in UC compared to controls. AGPAT1 is a potential colonic biomarker for distinguishing PSC-UC from UC.	[48]
UC	102 patients versus 156 controls	Serum	TAMBP, SIRT2, SCAMP3, CD5, ADAM8, GZMB, MMP-10, CXCL9, CDKN1A, CCL11, ABL1, TNFRSF6B	Increased	The protein panel has a superior ability to discriminate between UC patients and the control group (AUC = 0.95; 95% CI: 0.92–0.99).	[49]
CD	54 patients versus 156 controls	Serum	CXCL9, IL-6, MMP-10, CCL20, MDK, CXCL17	Increased	Increased ability to differentiate CD patients from controls (AUC = 0.85; 95% CI: 0.78–0.93).	[49]
			DNER, GPNMB, CX3CL1	Decreased		
IBD	328 patients versus 224 controls	Serum	MMP-12, OSM, CXCL1, IL-8, IL-17A, CXCL9, GrB, MMP-10, CXCL11, HGF	Increased	The model differentiates IBD from the control group (accuracy = 0.798, 95% CI: 0.764–0.832; sensitivity = 0.831, 95% CI: 0.791–0.872; specificity = 0.748, 95% CI: 0.690–0.805).	[43]
			GAS6, ITGAV	Decreased		
IBD	24 patients versus 9 controls	Colonic tissue	CHI3L1, PNP, OLFM4, LCN2, MMP9, NAMPT, NNMT, PARP9, PARP14, NFKB2, CD38, S100A12,	Increased	The model differentiates IBD patients from the control group.	[50]
			ITLN1, NNT, NT5C3A, NADK2	Decreased		
IBD	60 patients (30 UC, 30 CD) versus 39 controls	Colonic tissue	FABP5, UGDH, Visfatin, LRPPRC, PPA1	Increased	The model proved superior accuracy to distinguish IBD patients from healthy controls (AUC = 1.0, 95% CI: 0.99–1.0; precision = 0.95, 95% CI: 0.86–1.0; sensitivity = 1.0, 95% CI: 0.83–1.0; specificity = 0.93, 95% CI: 0.78–0.99).	[51]
			HADHB, LAP3, LTAH4, MT2, B2M, TRFC, SL25A1, ECH1, HNRNPH3	Increased	The panel of 12 proteins differentiated patients with CD from UC (AUC = 0.95; 95% CI: 0.86–1.0; accuracy = 0.80; sensitivity = 1.0, 95% CI: 0.78–1.0; specificity = 0.93, 95% CI: 0.68–1).	
			SEC61A1, SND1, TF	Decreased		
IBD	83 patients (27 UC, 56 CD) versus 12 controls	Serum	GUC2A, CHGB	Increased	UC could be differentiated from CD by elevated levels of GUC2A (AUC = 0.80, specificity = 0.89, sensitivity = 0.67 *p* = 0.0006) and CHGB (AUC = 0.70, specificity = 0.78, sensitivity = 0.67, *p* = 0.008).	[52]
IBD	43 patients (22 UC versus 21 CD)	Colonic tissue	ATP1A1, HIST	Increased	The variability of protein signatures in intestinal epithelial cells distinguishes between CD and UC.	[53]
			MYH9, MVP, HIST1H2AC	Decreased		
IBD	117 patients (57 UC, 60 CD) versus 31 controls	Feces	GSN, RhoGDI2	Increased	The two proteins have a much better performance to differentiate CD patients from the control group GSN, (AUC = 0.998, sensitivity = 0.91, specificity = 1.0, *p* = 0.0009; RhoGDI2 (AUC = 1.0, sensitivity = 1.0, specificity = 1.0, *p* = 0.0004) compared to FC (AUC = 0.824, sensitivity = 0.86, specificity = 0.68).	[54]
			RhoGDI2	Increased	This protein has maximum precision (AUC = 1, sensitivity = 1.0, specificity = 1.0, *p* = 0.0009) for discriminating UC patients from healthy controls.	
IBD	24 patients (12 UC, 12 CD) versus 9 controls	Colonic tissue	ALDOB, FABP2, ACE1, ACE2, S100A8, S100A9, MPO, LTF	Increased	Upregulation of protein expression was observed in CD compared to UC.	[50]
IBD	121 patients (71 CD, 60 UC) versus 10 controls	Colonic tissue	KRT4, ELANE, S100A9, S100A8, CTSG, LTF, LYZ, IGHM, AKR1C3, AKR1C1	Increased	Each of these proteins is expressed at levels at least three times higher in CD patients compared to UC. AKR1C3 and AKR1C1 were expressed exclusively in CD patients.	[55]
IBD	76 patients (30 UC, 30 CD) versus 16 controls	Plasma	Resistin, Elastase	Increased	Circulating resistin is significantly increased in UC (AUC = 0.82, sensitivity = 0.77, specificity = 0.88) and CD (AUC = 0.77, sensitivity = 0.70, specificity = 0.88) (*p* < 0.01). Increased levels of elastase are detected in UC (AUC = 0.74, sensitivity = 0.57, specificity = 0.94).	[56]
IBD	193 patients (118 UC, 75 CD) versus 32 controls	Serum	VICM, C3M, C4M	Increased	The combinations of proteins used for discrimination between CD from UC, and UC from martors are VICM, C3M, C4M (AUC = 0.86, specificity = 0.90, sensitivity = 0.75, accuracy = 0.79) and VICM, C3M (AUC = 0.98, specificity = 0.94, sensitivity = 0.96, accuracy = 0.95), respectively.	[57]

UC: Ulcerative colitis; CD: Crohn’s disease; IBD: Inflammatory bowel disease; LTF: Lactotransferrin; MMP-9: Matrix Metalloproteinase-9; MPO: Myeloperoxidase; NE: Neutrophil Elastase; S100-A9: Protein S100-A9; S100-A12: Protein S100-A12; DEF3: Neutrophil Defensin 3; Gal-10: Galectin-10; ECP: Eosinophil Cationic Protein; MNDA: Myeloid Cell Nuclear Differentiation Antigen; CatG: Cathepsin G; NETs: Neutrophil Extracellular Traps; ABCB1: ATP Binding Cassette Subfamily B Member 1; ABCG2: ATP Binding Cassette Subfamily G Member 2; MCT1: Monocarboxylate Transporter 1; AGPAT1: 1-Acylglycerol-3-Phosphate O-Acyltransferase 1; CD47: Leukocyte Surface Antigen CD47; NDUFAF4: NADH:Ubiquinone Oxidoreductase Complex Assembly Factor 4; TMEM192: Transmembrane Protein 192; LSM-7: U6 snRNA-Associated Sm-Like Protein LSm7; PSC-UC: Primary Sclerosing Cholangitis-Associated Ulcerative Colitis; MMP-10: Matrix Metalloproteinase-10; MCP-1: Monocyte Chemoattractant Protein-1; CXCL9: C-X-C Motif Chemokine Ligand 9; CCL11: C-C Motif Chemokine Ligand 11; SLAMF1: Signaling Lymphocytic Activation Molecule Family Member 1; CXCL11: C-X-C Motif Chemokine Ligand 11; AUC: Area Under the Curve; TAMBP: Tamm-Horsfall Glycoprotein Binding Protein; SIRT2: Sirtuin 2; SCAMP3: Secretory Carrier-Associated Membrane Protein 3; ADAM8: A Disintegrin and Metalloproteinase Domain-Containing Protein 8; GZMB: Granzyme B; ABL1: Abelson Tyrosine-Protein Kinase 1; CD5: T-cell Surface Glycoprotein CD5; TNFRSF6B: Tumor Necrosis Factor Receptor Superfamily Member 6B; GrB; Granzyme B; CDKN1A: Cyclin-Dependent Kinase Inhibitor 1A; IL-6: Interleukin-6; CCL20: C-C Motif Chemokine Ligand 20; MDK: Midkine; CXCL17: C-X-C Motif Chemokine Ligand 17; DNER: Delta and Notch-Like Epidermal Growth Factor-Related Receptor; GPNMB: Glycoprotein NMB; CX3CL1: C-X3-C Motif Chemokine Ligand 1; MMP-12: Matrix Metalloproteinase-12; OSM: Oncostatin M; CXCL1: C-X-C Motif Chemokine Ligand 1; IL-8: Interleukin-8; IL-17A: Interleukin-17A; HGF: Hepatocyte Growth Factor; GAS6: Growth Arrest-Specific Protein 6; ITGAV: Integrin Subunit Alpha V; CHI3L1: Chitinase 3-Like Protein 1; OLFM4: Olfactomedin-4; CD38: ADP-Ribosyl Cyclase 1; LCN2: Lipocalin-2; PNP: Purine Nucleoside Phosphorylase; NAMPT: Nicotinamide Phosphoribosyltransferase; NNMT: Nicotinamide N-Methyltransferase; PARP9: Poly (ADP-Ribose) Polymerase Family Member 9; PARP14: Poly(ADP-Ribose) Polymerase Family Member 14; NFKB2: Nuclear Factor Kappa B Subunit 2; ITLN1: Intelectin-1; NT5C3A: 5′-Nucleotidase, Cytosolic IIIA; NADK2: NAD Kinase 2, Mitochondrial; NNT: Nicotinamide Nucleotide Transhydrogenase; FABP5: Fatty Acid-Binding Protein 5; UGDH: UDP-Glucose 6-Dehydrogenase; Visfatin: Nicotinamide Phosphoribosyltransferase; LRPPRC: Leucine-Rich Pentatricopeptide Repeat Motif-Containing Protein; PPA1: Inorganic Pyrophosphatase; SEC61A1: Protein Transport Protein Sec61 Subunit Alpha isoform 1; SND1: Staphylococcal Nuclease Domain-Containing Protein 1; TF: Serotransferrin; GUC2A: Guanylin; CHGB: Secretogranin-1; ATP1A1: Sodium/Potassium-Transporting ATPase Subunit Alpha-1; HIST: Histone Protein; MYH9: Myosin Heavy Chain 9; MVP: Major Vault Protein; HIST1H2AC: Histone Cluster 1 H2A Family Member C; GSN: Gelsolin; RhoGDI2: Rho GDP-Dissociation Inhibitor 2; ALDOB: Fructose-Bisphosphate Aldolase B; FABP2: Fatty Acid-Binding Protein 2; ACE1: Angiotensin-Converting Enzyme 1; ACE2: Angiotensin-Converting Enzyme 2; S100A8: Protein S100-A8; KRT4: Keratin 4; ELANE: Neutrophil Elastase; CTSG: Cathepsin G; LYZ: Lysozyme; IGHM: Immunoglobulin Heavy Constant Mu; AKR1C3: Aldo-Keto Reductase Family 1 Member C3; AKR1C1: Aldo-Keto Reductase Family 1 Member C1; VICM: Matrix Metalloproteinase-Degraded and Citrullinated Vimentin; C3M: Matrix Metalloproteinase-Degraded Collagen Type III; C4M: Matrix Metalloproteinase-Degraded Collagen Type IV.

**Table 3:** Proteomics in monitoring the evolution of IBD.

Disease	Number of Patients	Sample	Main Findings	References
**UC**	10 patients versus 10 controls	Colonic tissue	Strong correlation between the severity of inflammatory lesions and the presence of specific tissue proteins (S100-A8, *r* = 0.84; S100-A12, *r* = 0.91; LF, *r* = 0.82).	[41]
**UC**	64 patients versus 47 controls	Colonic tissue	MUC2 and SLC26A3 were significantly reduced in non-inflamed intestinal segments (*p* < 0.0001). The reduction in mucus-associated SLC26A3 levels was particularly pronounced in individuals in remission.	[66]
**UC**	19 patients	Colonic tissue	Neutrophil-related proteins (MPO, ELANE, CD44, CD55, CYBA, CYBB, CAM1, ITGAM, ITGB2, and MMP9) correlate with GS scores (sensitivity: 72.7%, specificity: 100%) and RHI index (sensitivity: 75%, specificity: 81.8%) in active UC.	[65]
**UC**	51 patients versus 17 controls	Colonic tissue	Up-regulation of the tissue proteins TRX (AUC = 0.91, 95% CI: 0.79–100; sensitivity = 86%, specificity = 85%, accuracy = 85%) and IGHA (AUC = 0.89, 95% CI: 0.75–1.00; sensitivity = 71%, specificity = 85%, accuracy = 80%) is predictive for early recurrence.	[68]
**CD**	64 patients	Serum	A combination of three proteins (DSG1, DSP, and FABP5) released from transmural intestinal lesions is predictive of complications in CD (AUC = 0.777, sensitivity = 70.0%, specificity = 72.5%, *p* = 0.007).	[69]
**CD**	20 patients	Serum	A panel of five serum glycoproteins (COMP, HGFA, POCE, Che, TNXB) showed 20% to 80% higher abundance in CD patients with stenotic complications compared to those with the inflammatory phenotype.	[62]
**CD**	102 patients	Serum	Linked to elevated serum levels of lymphocyte-expressed proteins (LAG3, SH2B3, SIT1; HR: 2.2–4.5) and decreased concentrations of anti-inflammatory effectors (IL-10, HSD11B1; HR: 0.2–0.3) and cell junction proteins (CDSN, CNTNAP2, CXADR, ITGA11; HR: 0.4) is associated with long-term risk of relapse (*p* < 0.05).	[60]
**CD**	30 patients versus 15 controls	Serum	The combination of three proteins (WDR3, LRG1, and SAA1) predicts progression in CD patients with a stricturing or penetrating phenotype (AUC = 0.737).	[37]
**CD**	589 patients	Serum	The EHI includes 13 serum proteins (ANG1, ANG2, CRP, SAA1, IL7, EMMPRIN, MMP1, MMP2, MMP3, MMP9, TGFA, CEACAM1, and VCAM1) for predicting remission in CD patients (AUC = 0.962; sensitivity = 0.971; specificity = 0.690).	[58]
**CD**	116 patients	Serum	ECM1 (HR = 3.41, 95% CI: 1.33–8.42; *p* < 0.001), IgA ASCA (HR = 4.99, CI 95%: 1.50–16.68) and CBir (HR = 5.19, CI 95%: 1.83–14.74) represents a predictive biomarkers for the development of colonic strictures in pediatric CD patients.	[70]
**CD**	161 patients versus 40 controls	Plasma	Elevated COL3A1 and anti-CSF2 concentrations at the time of diagnosis are predictive for the occurrence of stenotic complications in pediatric patients (AUC = 0.8, CI 95%, 0.71–0.89; sensitivity = 0.7, CI 95%, 0.55–0.83; specificity = 0.83, CI 95%, 0.67–0.93).	[71]
**CD**	265 patients	Serum	A model including five proteins (NFSF14, CCL4, IL15RA, TNFB, and CD40) expressed in ileal T cells and peripheral blood predicted the occurrence of penetrating complications more effectively (AUC = 0.79) compared to serological markers (LnASCA IgA, LnANCA, LnCbir) (AUC = 0.69) and clinical variables (AUC = 0.74).	[72]
**CD**	73 patients versus 40 controls	Colonic tissue	The development of fibrotic strictures in CD patients is associated with the hypersecretion of BiP and AGR2 in colonic epithelium.	[73]
**CD**	112 patients versus 24 controls	Serum	Serum levels of collagen formation and degradation products (P1NP, Pro-C3, Pro-C5, Pro-C6, C1M, C3M, C5M, and C6M) are higher in patients with active endoscopic inflammation. Pro-C3 and C3M present the greatest potential for differentiating penetrating vs. non-penetrating CD (AUC = 0.815, *p* < 0.001) and stricturing disease (AUC = 0.746, *p* = 0.002).	[74]
**CD**	101 patients versus 96 controls	Serum	Increased degradation of collagen markers C1M, C3M, and C4G is significantly associated with the development of strictures (HR: 1.71; *p* < 0.05). Higher baseline concentrations of C1M and C4G were linked to an elevated risk of progression to the penetrating form of the disease (HR: 1.71; 95% CI: 1.05–2.81; *p* < 0.05).	[75]
**IBD**	143 patients (39 UC, 104 CD) versus29 controls	Serum	ELP-3 is specific to UC with an active clinical phenotype (AUC = 0.870; sensitivity = 83.3%; specificity = 76.2%; *p* < 0.0001), while ELM-12 is significantly elevated in CD patients in endoscopic remission (AUC = 0.73; sensitivity = 94.4%; specificity = 51.2%; *p* = 0.001).	[76]
**IBD**	117 patients (60 UC, 57 CD) versus 31 controls	Feces	Three fecal protein markers were significantly correlated with the severity of intestinal inflammation in CD (CTRC: *r* = 0.64, *p* < 0.001; GSN: *r* = 0.82, *p* < 0.001; RhoGDI2: *r* = 0.64, *p* < 0.001) and UC (CTRC: *r* = 0.76, *p* < 0.001; GSN: *r* = 0.75, *p* < 0.001; RhoGDI2: *r* = 0.63, *p* < 0.001).	[54]
**IBD**	60 patients (30 UC, 30 CD) versus 29 controls	Colonic tissue	Visfatin and MT2 are significantly correlated with the severity of clinical progression in CD (r = 0.5186, *p* = 0.0025; r = 0.5975, *p* = 0.007, respectively), while in UC, an opposite relationship was observed with HNRNP-H3 (r = −0.2791, *p* = 0.035).	[51]

**UC**: Ulcerative colitis; **CD**: Crohn’s disease; **IBD**: Inflammatory bowel disease; **S100-A8**: Protein S100-A8; **S100-A12**: Protein S100-A12; **LF**: Lactoferrin; **MUC2**: Mucin 2; **SLC26A3**: Solute Carrier Family 26 Member 3; **MT2**: Metallothionein-2; **HNRNP-H3**: Heterogeneous Nuclear Ribonucleoprotein H3; **MPO**: Myeloperoxidase; **ELANE**: Neutrophil Elastase; **CD44**: Cluster of Differentiation 44; **CD55**: Cluster of Differentiation 55; **CYBA**: Cytochrome b-245 Alpha Chain; **CYBB**: Cytochrome b-245 Beta Chain; **CAM1**: Cell Adhesion Molecule 1; **ITGAM**: Integrin Subunit Alpha M; **ITGB2**: Integrin Subunit Beta 2; **MMP9**: Matrix Metalloproteinase-9; **GS**: Geboes Score; **RHI**: Robarts Histopathology Index; **DSG1**: Desmoglein-1; **DSP**: Desmoplakin; **FABP5**: Fatty Acid Binding Protein 5; **AUC**: Area Under the Curve; **COMP**: Cartilage Oligomeric Matrix Protein; **HGFA**: Hepatocyte Growth Factor Activator; **POCE**: Procollagen C-Endopeptidase Enhancer; **Che**: Cholinesterase; **TNXB**: Tenascin XB; **CTRC**: Chymotrypsin-C; **GSN**: Gelsolin; **HR**: Hazard Ratio; **IL-10**: Interleukin 10; **HSD11B1**: Hydroxysteroid 11-Beta Dehydrogenase 1; **CDSN**: Corneodesmosin; **CNTNAP2**: Contactin-Associated Protein-Like 2; **CXADR**: Coxsackievirus and Adenovirus Receptor; **ITGA11**: Integrin Subunit Alpha 11; **ANG1**: Angiopoietin 1; **ANG2**: Angiopoietin 2; **CRP**: C-Reactive Protein; **SAA1**: Serum Amyloid A1; **IL7**: Interleukin 7; **EMMPRIN**: Extracellular Matrix Metalloproteinase Inducer; **MMP1**: Matrix Metalloproteinase-1; **MMP2**: Matrix Metalloproteinase-2; **MMP3**: Matrix Metalloproteinase-3; **MMP9**: Matrix Metalloproteinase-9; **TGFA**: Transforming Growth Factor Alpha; **CEACAM1**: Carcinoembryonic Antigen-Related Cell Adhesion Molecule 1; **VCAM1**: Vascular Cell Adhesion Molecule 1; **WDR3**: WD Repeat Domain 3; **LRG1**: Leucine-Rich Alpha-2-Glycoprotein 1; **ECM1**: Extracellular Matrix Protein 1; **NFSF14**: Tumor Necrosis Factor Superfamily Member 14; **CCL4**: C-C Motif Chemokine Ligand 4; **IL15RA**: Interleukin 15 Receptor Alpha; **TNFB**: Tumor Necrosis Factor Beta; **CD40**: Cluster of Differentiation 40; **BiP**: Binding Immunoglobulin Protein; **AGR2**: Anterior Gradient 2; **P1NP**: Procollagen Type 1 N-Terminal Propeptide; **Pro-C3**: Procollagen Type III N-Terminal Propeptide; **Pro-C5**: Procollagen Type V N-Terminal Propeptide; **Pro-C6**: Procollagen Type VI N-Terminal Propeptide; **C1M**: MMP-degraded Type I Collagen; **C3M**: MMP-degraded Type III Collagen; **C5M**: MMP-degraded Type V Collagen; **C6M**: MMP-degraded Type VI Collagen; **C4G**: Complement Component 4 Gamma Chain; **ELP-3**: Elastin Degradation Product 3; **ELM-12**: Elastin Degradation Marker 12.

**Table 4:** Proteomics involved in Therapeutic Response in IBD.

Disease	Medication	Proteins	Sample	Main Findings	References
**UC**	**IFX**	**TNC, CCL2**	Serum Colonic tissue	A favorable response is associated with downregulation in tissue levels of TNC and serum expression of CCL2.	[85]
**UC**	**IFX**	**ACTBL2, MBL2, BPI, EIF3D, CR1**	Colonic tissue	Potential biomarkers of non-response to IFX therapy.	[92]
**UC**	**VDZ**	**OC**	Serum	Expression is increased in responders (sensitivity 85%, specificity 100%).	[93]
**UC**	**VDZ**	**s-** **α** **4** **β** **7, s-TNF, s-MAdCAM-1, s-VCAM-1, s-ICAM-1**	Serum	An increase in serum s-α4β7 levels accompanied by a decrease in s-MAdCAM-1, s-VCAM-1, s-ICAM-1, and s-TNF concentrations was observed in patients with endoscopic remission.	[94]
**UC**	**IFX** **ADA**	**NGAL-MMP-9, CHI3L1, CRP, LL-37**	Serum	A significant reduction in serum proteins and neutrophil count (UCRI index) accurately detects MH, after IFX (AUC = 0.83) and ADA (AUC = 0.79).	[95]
**CD**	**IFX** **ADA**	**C4M**	Serum	Patients with elevated baseline serum levels of C4M do not respond to IFX (OR = 39; sensitivity 0.93, specificity 0.75, *p* = 0.02) or ADA (OR = 26; sensitivity 0.93, specificity 0.67, *p* = 0.01).	[74]
**CD**	**IFX**	**PF4**	Serum	Higher levels were found in non-responders.	[84]
**CD**	**IFX**	**PRO-C3, PRO-C6, C4M**	Serum	C4M discriminates CD patients with a history of surgery into responders versus non-responders before IFX treatment (AUC = 0.84; *p* = 0.016). PRO-C3 and PRO-C6-used for monitoring therapeutic efficacy (AUC = 0.95; *p* = 0.004, and AUC = 0.82; *p* = 0.037).	[89]
**CD**	**VDZ**	**sCD40L**	Serum	An increase in serum concentration is predictive of therapeutic non-response (sensitivity 100%, specificity 100%).	[93]
**CD**	**IFX**	**MMP3, CRP, CCL2**	Serum	The combined model measured at week 2 of treatment proves excellent performance (AUC = 0.898) in predicting primary non-response.	[94]
**CD**	**VDZ**	**s-MAdCAM-1, s-VCAM-1, s-ICAM-1, s-** **α** **4** **β** **7**	Serum	Increased levels of s-ICAM-1 and s-VCAM-1 are predictive of endoscopic remission. In responders, a significant reduction in serum MAdCAM-1 concentration, while s-α4β7 levels are increased.	[96,97]
**CD**	**VDZ**	**C1M, CPa9-HNE, C6Ma3, C3M, C4M, PRO-C3, PRO-C4**	Serum	Serological biomarkers of extracellular matrix turnover and neutrophil activity have significantly increased baseline concentrations in non-responders.	[98]
**CD**	**IFX**	**VTDB** **,** **A1BG** **, C1R, A2GL**	Serum	Identification of proteins with increased abundance in infliximab-induced clinical and serological remission compared to baseline samples.	[83]
		**APOA1, CLUS, APOE, APOH, CO4B, TRFE, PLMN**		Increased serum expression of these proteins in non-responding patients.	
**IBD**	**IFX** **ADA**	**ITGAV, IL-8, IL-18, EpCAM, SLAMF7**	Serum	The overexpression is predictive of biologic therapy escalation or the necessity for surgical intervention (HR = 3.9; 95% CI: 2.43–6.26).	[43]
**IBD**	**IFX**	**TNC**	Colonic tissue	Increased expression of TNC in inflamed intestinal mucosa was associated with a reduced response to IFX therapy.	[50]
**IBD**	**IFX**	**IL-8, HGF, 4E-BP1, MCP-3, MMP-10, OSM, TGF-α**	Serum	Non-responding patients—elevated baseline serum concentrations of seven proteins at the initiation of induction therapy.	[81]
**IBD**	**IFX** **ADA**	**MMP3, MMP12**	Serum	Serum levels of endogenous IgG cleaved by MMP3 and MMP12 were higher in non-responders.	[82]
**IBD**	IFX CS	**SERPINA1, CCL23, IGFBP1, IGFBP2, RETNi**	Serum	These proteins with inflammatory functions showed significant reductions post-therapy.	[99]
**IBD**	**VDZ**	**α4β7**	Serum	Higher expression of α4β7 on T effector memory cells and NK cells is predictive of a favorable response.	[100]

**UC**: Ulcerative colitis; **CD**: Crohn’s disease; **IBD**: Inflammatory bowel disease; **IFX**: Infliximab; **ADA**: Adalimumab; **IBD**: Inflammatory Bowel Disease; **UC**: Ulcerative Colitis; **CD**: Crohn’s Disease; **VDZ**: Vedolizumab; **PF4**: Platelet Factor 4; **VTDB**: Vitamin D-Binding Protein; **A1BG**: Alpha-1B-Glycoprotein; **MBL2**: mannose-binding protein C; **BPI**: bactericidal permeability-increasing protein; **EIF3D**: Eukaryotic translation initiation factor-3; **CR1**: Complement C1r Subcomponent; **A2GL**: Alpha-2-Glycoprotein-like; **APOA1**: Apolipoprotein A1; **APOE**: Apolipoprotein E; **CO4B**: Complement Component 4B; **PLMN**: Plasminogen; **TRFE**: Transferrin; **APOH**: Apolipoprotein H; **CLUS**: Clusterin; **TNC**: Tenascin C; **CCL2**: C-C Motif Chemokine Ligand 2; **MMP3**: Matrix Metalloproteinase-3; **MMP12**: Matrix Metalloproteinase-12; **SERPINA1**: Serpin Family A Member 1; **IGFBP1**: Insulin-like Growth Factor-Binding Protein 1; **IGFBP2**: Insulin-like Growth Factor-Binding Protein 2; **ACTBL2**: Actin Beta Like 2; **RETNi**: Resistin; **CCL23**: C-C Motif Chemokine Ligand 23; **sCD40L**: Soluble CD40 Ligand; **OC**: Osteocalcin; **α4β7**: Integrin Alpha-4 Beta-7; **s-α4β7**: Soluble α4β7; **s-MAdCAM-1**: Soluble Mucosal Addressin Cell Adhesion Molecule-1; **s-VCAM-1**: Soluble Vascular Cell Adhesion Molecule-1; **s-ICAM-1**: Soluble Intercellular Adhesion Molecule-1; **s-TNF**: Soluble Tumor Necrosis Factor; **NGAL-MMP-9**: Neutrophil Gelatinase-Associated Lipocalin and Matrix Metalloproteinase-9 Complex; **CHI3L1**: Chitinase-3-Like Protein 1; **CRP**: C-Reactive Protein; **LL-37**: Cathelicidin Antimicrobial Peptide; **C4M**: MMP-degraded Type IV Collagen; **ITGAV**: Integrin Subunit Alpha V; **EpCAM**: Epithelial Cell Adhesion Molecule; **IL-18**: Interleukin 18; **SLAMF7**: SLAM Family Member 7; **IL-8**: Interleukin 8; **PRO-C3**: Procollagen Type III N-Terminal Propeptide; **PRO-C6**: Procollagen Type VI N-Terminal Propeptide; **C1M**: MMP-degraded Type I Collagen; **C3M**: MMP-degraded Type III Collagen; **C6Ma3**: MMP-degraded Type VI Collagen Neo-Epitope; **PRO-C4**: Procollagen Type IV N-Terminal Propeptide; **CPa9-HNE**: Calprotectin Peptide Associated with Human Neutrophil Elastase Activity; **HGF**: Hepatocyte Growth Factor; **4E-BP1**: Eukaryotic Translation Initiation Factor 4E-Binding Protein 1; **MCP-3**: Monocyte Chemoattractant Protein-3; **OSM**: Oncostatin M; **TGF-α**: Transforming Growth Factor Alpha; **MMP-10**: Matrix Metalloproteinase-10.

## Data Availability

No new data were created or analyzed in this study.
